# Effect of Ultrasonication Parameters on the Structural, Morphological, and Electrical Properties of Polypyrrole Nanoparticles and Optimization by Response Surface Methodology

**DOI:** 10.3390/polym15061528

**Published:** 2023-03-20

**Authors:** SK Safdar Hossain, Anis Farhana Abdul Rahman, Agus Arsad, Avijit Basu, Ai Ling Pang, Zakiah Harun, Muhammad Mudassir Ahmad Alwi, Syed Sadiq Ali

**Affiliations:** 1 Department of Chemical Engineering, King Faisal University, Al-Ahsa 31982, Saudi Arabia; snooruddin@kfu.edu.sa (S.S.H.);; 2 UTM-MPRC Institute for Oil and Gas, Faculty of Chemical and Energy Engineering, Universiti Teknologi Malaysia, Johor Bahru 81310, Malaysia; 3 Department of Chemical Science, Faculty of Science, Universiti Tunku Abdul Rahman, Kampar 31900, Malaysia; 4 Department of Materials Engineering, King Faisal University, Al-Ahsa 31982, Saudi Arabia

**Keywords:** polypyrrole nanoparticles, ultrasonication, sonication parameters, response surface methodology, conductivity

## Abstract

Polypyrrole (PPy) nanoparticles are reliable conducting polymers with many industrial applications. Nevertheless, owing to disadvantages in structure and morphology, producing PPy with high electrical conductivity is challenging. In this study, a chemical oxidative polymerization-assisted ultra-sonication method was used to synthesize PPy with high conductivity. The influence of critical sonication parameters such as time and power on the structure, morphology, and electrical properties was examined using response surface methodology. Various analyses such as SEM, FTIR, DSC, and TGA were performed on the PPy. An R^2^ value of 0.8699 from the regression analysis suggested a fine correlation between the observed and predicted values of PPy conductivity. Using response surface plots and contour line diagrams, the optimum sonication time and sonication power were found to be 17 min and 24 W, respectively, generating a maximum conductivity of 2.334 S/cm. Meanwhile, the model predicted 2.249 S/cm conductivity, indicating successful alignment with the experimental data and incurring marginal error. SEM results demonstrated that the morphology of the particles was almost spherical, whereas the FTIR spectra indicated the presence of certain functional groups in the PPy. The obtained PPy with high conductivity can be a promising conducting material with various applications, such as in supercapacitors, sensors, and other smart electronic devices.

## 1. Introduction

Conductive polymers such as polyaniline, polypyrrole (PPy), polythiophene, and their derivatives are receiving extensive attention owing to their outstanding optical and electronic properties [1,2] and applications in different areas, specifically as potential hybrid materials [3,4]. In fact, a polymeric material is ideal for shielding external electromagnetic interference because of its low cost, light weight, corrosion resistance, ease of processing, and tunable conductivity [5].

Among the known conducting polymers, PPy is extensively used in commercial applications owing to its high conductivity, high energy density, good durability, and low toxicity [6,7]. PPy is extensively used in many applications, including electrocatalysts, sensors, rechargeable batteries, and biomedicines [8,9,10]. The structure and conductivity of polymers play important roles in determining their efficiency in various applications. However, preparing conductive polymers with conventional blending or mixing in solution is difficult because the conductive polymers are hard to dissolve in common solvents and easily aggregate owing to their high surface energy and strong interactions [11]. Thus, the synthesis method is a crucial parameter that plays an important role in producing profoundly structured and highly conductive polymers.

The most extensively used method for PPy synthesis is chemical oxidative polymerization in the presence of anionic surfactants such as sodium dodecyl sulfate and sodium dodecylbenzenesulfonate to accelerate the polymerization [12,13]. Many oxidants have also been used in typical chemical oxidative polymerization for PPy synthesis, including ferric chloride, ferric perchlorate, ammonium peroxydisulfate, and others [14,15]. The properties of fabricated conducting polymers are affected by various additives introduced into reaction mixtures and synthesis conditions. For instance, Wu et al. [16] conducted in situ chemical oxidative polymerization with ferric chloride as the oxidant to synthesize PPy with high conductivity using various concentrations of cetyltrimethylammonium bromide. However, this method has the drawbacks of difficulty in obtaining homogeneous PPy with a uniform structure and difficulty in synthesizing rigid insoluble polymers [17].

Apart from chemical oxidative polymerization, electrochemical oxidative polymerization is also widely used to synthesize PPy. Qu et al. [18] synthesized PPy using electrochemical oxidative polymerization, in which the pyrrole (Py) monomer was oxidized on the electrode surface in an electrolyte solution. Nevertheless, this method is generally suitable only for small-scale synthesis owing to the need for specialized equipment, such as working and reference electrodes, which can make scaling up the synthesis difficult and costly [12,17]. This method may also undergo overoxidation, leading to unwanted processes such as electrolysis product formation or solvent oxidation. The consequences are impurities and reduced quality of the synthesized polymer.

Meanwhile, ultrasonication is a promising approach with a rapid reaction rate, greener method, uniform shape, purity of prepared samples, and smaller particles than with other methods [19,20,21]. Ultrasonication is also known to be effective in modifying the structural and morphological properties of polymers, resulting in a significantly increased conductivity [22]. The use of ultrasonication aids the chemical oxidative and electrochemical polymerization of PPy [23]. Ultrasonication involves the application of high shear forces to the mixture and the creation of bubbles with ultrasonic waves within a medium. The bubbles grow until they collapse violently when high- and low-pressure waves impinge on them [24]. For instance, Kowalski et al. [23] imposed ultrasonic irradiation during PPy electropolymerization. They found that the initial stage of polymerization has a higher number of nuclei under ultrasonic irradiation compared with those without sonication, thereby enhancing the conductivity of PPy. Moreover, Li et al. [8] studied the effect of methyl orange (MO) and ethyl orange (EO) dyes on PPy fabrication using ultrasonication-assisted chemical oxidative polymerization. In the present work, Py and FeCl_3_ solutions were blended in the presence of MO and EO dyes. After ultrasonication for 2–3 min, the reaction was left undisturbed for 24 h. The conductivity of PPy nanotubes prepared with MO and EO increased to 92.5 and 6.8 S/cm, respectively, indicating that the existence of dyes could greatly affect the electrical conductivity of PPy thin films.

The most important parameters to be controlled during ultrasonication are sonication power and time because they can have crucial effects on the properties of the synthesized polymer. When the sonication is too vigorous or too long, the polymers could be damaged, which could in turn affect the process efficiency. Yang et al. [25] prepared PPy under different ultrasonication powers. They discovered that with increased ultrasonic power to >44 W, PPy conductivity decreases, and the chain structure and quality of PPy deteriorate at extremely high ultrasonic power. Mohsin et al. [26] performed several experiments at various sonication times of 0.5, 2, 3, and 5 h. They discovered that the electrical conductivity of blend polymers varies with time, and the highest electrical conductivity is obtained at 2 h of sonication time. Sonication for less than 2 h results in low electrical conductivity owing to insufficient time for monomer oxidization. Meanwhile, prolonging the sonication time by >2 h increases the energy in the sonication bath, leading to polymer collapse.

To the best of our knowledge, there are few studies on the effect of more parameters. Researchers have focused only on the effect of one parameter individually and kept the other parameter constant. To evaluate more parameters for the synthesis PPy, the design of experiments (DOE) method is suitable [27]. This method can optimize process parameters and reduce the number of experiments to achieve high electrical conductivity with low resistivity. Among the DOE methods, the response surface methodology (RSM) is an effective tool for analyzing changes in process response by varying the experimental variables at the same time and performing a limited number of experiments from which quadratic models can be derived. Accordingly, the current work investigated the effects of sonication power and sonication time on PPy synthesis using ultrasonic irradiation-assisted in situ polymerization. The influence of both sonication parameters on the structural, morphological, thermal, and electrical conductivity was investigated. To achieve optimum ultrasonication parameters, RSM was used.

## 2. Materials and Methods

### 2.1. Materials

Py, FeCl_3_, and ethanol were purchased from Sigma–Aldrich (M) Sdn. Bhd. The molecular weights of Py, FeCl_3_, and ethanol were 67.09, 162.2, and 46.07 g/mol, respectively. HCl (37%) with a molecular weight of 36.46 g/mol was obtained from Qrec (Asia) Sdn. Bhd.

### 2.2. PPy Nanoparticle Preparations

**Figure 1:**
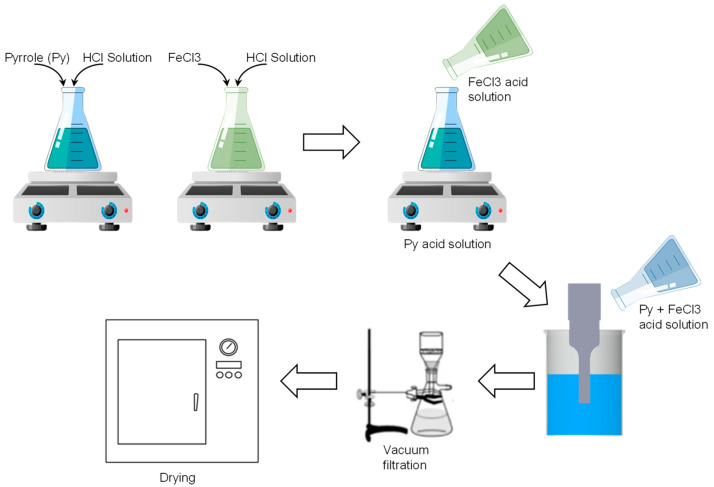
Schematic of PPy preparation using ultrasonication.

**Figure 2:**
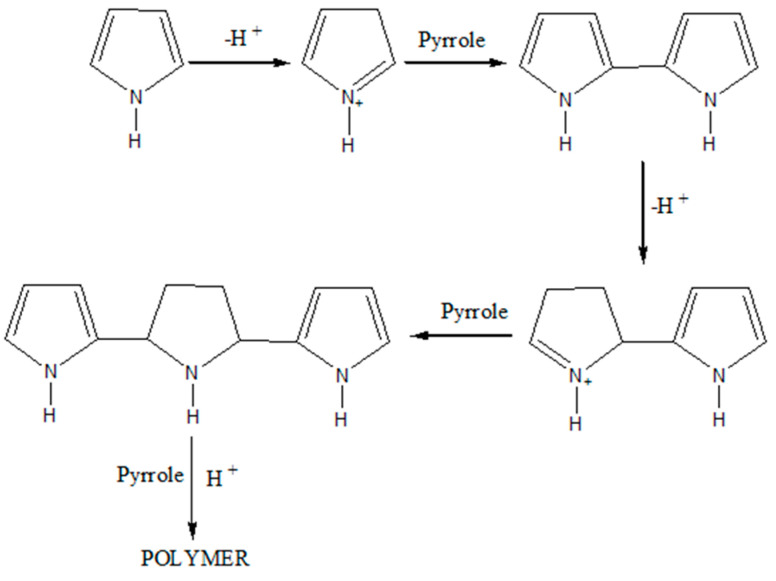
Schematic of polypyrrole mechanism [28].

PPy was prepared using ultrasonic-assisted chemical oxidative polymerization with FeCl_3_ as an oxidant and HCl as a dopant. First, Py and FeCl_3_ were mixed separately with 100 mL of HCl solution in a conical flask. The mixture was stirred continuously at a constant speed under atmospheric conditions for 30 min. The FeCl_3_ acid solution was added dropwise into the Py acid solution. Ultrasonic irradiation (Biobase Ultrasonic Cell Disrupter UCD 2000, Shandong, China) was then performed with the probe of the ultrasonic horn immersed directly into the mixture at a constant temperature (<10 °C) using an iced water bath to avoid any unwanted reactions. The frequency wave produced with the sonicator was constant at 30 kHz, with an on/off time of 1 s. The Py solution was ultrasonicated in accordance with the sonication parameters specified by RSM software. After ultrasonic irradiation, the obtained black-color solution was left undisturbed overnight for polymerization. Subsequently, the solution was filtered and washed with distilled water and methanol until the precipitate reached pH 7. The precipitate was dried overnight at 50 °C. Figure 1 and Figure 2 depict the process and proposed mechanism for the synthesis of PPy nanoparticles, respectively. As shown in Figure 2, the oxidant initially reacted with Py monomers to form a radical cation [28,29]. Then, two radical cations combined to form a dimer, and the process was repeated until longer chains formed. Basically, ultrasonic irradiation accelerated the oxidation, resulting in the formation of a homogeneous mixture of Py monomers and the oxidant, leading to more effective polymerization. The use of a terminating agent, which reacted with the radical cation, was required to stop the polymerization process.

### 2.3. Experimental Design and Optimization

A three-level-two-factor central composite design (CCD) was used to optimize the electrical conductivity of PPy. Sonication power (X_1_) and sonication time (X_2_) were selected as independent variables. The objective was to attain the electrical conductivity and resistivity of PPy, which was taken as the response. Table 1 provides the levels of the independent variables. Ten experiments were augmented with two duplications at the center points to assess the error. A quadratic model was fitted to the response variable (Y) after performing the experiments to correlate it with the independent variables. The influence of each variable, the sum of squares, mean square, *p*-value, F-value, and confidence level (%) were determined using face-centered CCD in statistical software to infer the optimum sonication parameter.

### 2.4. Characterization

The electrical conductivity of each sample was measured using a four-probe electrical conductivity tester (model ST2258C, Jiangsu, China). All samples were pressed into pellet form under 20 Mpa. Each composite pellet was measured five times to ensure accuracy, and the average conductivity values were reported. The data shown in Table 1 are the mean values of the measurements. Various methods were further used to characterize the samples, including scanning electron microscopy (SEM), Fourier transform infrared (FTIR) spectroscopy, thermogravimetric analysis (TGA), and differential scanning calorimetry (DSC). For the SEM analysis, XL 40; PW6822/10 (UK) was used to evaluate the morphology of PPy nanoparticles operated at 10 kV accelerating voltage. FTIR (PerkinElmer) was used to determine the functional groups of the samples at room temperature within the range of 400–4000 cm^−1^. Prior to scanning, an appropriate amount of the samples was mixed with KBr (IR grade) and pelletized. DSC studies were performed using Perkin Elmer DSC-6 at a heating rate of 10 °C/min from 30 °C to 500 °C under N_2_ atmosphere. To analyze the thermal stability of the produced PPy samples, TGA was performed using a Perkin Elmer 4000. Under N_2_ atmosphere, a sample weight of around 10 mg was placed in an alumina ceramic bowl and heated from 25 °C to 800 °C at a heating rate of 10 °C/min.

## 3. Results and Discussion

### 3.1. RSM Analysis

RSM is an approach for the determination of the optimum process conditions that allow users to obtain large amounts of information from a small number of experiments. Moreover, examining the relationships between variables and responses was possible, and it has been successfully applied to wide-ranging chemical reactions with multiple responses [30,31]. Table 1 shows the experimental CCD test matrix and the resulting analysis, which were conductivity and resistivity. Based on the RSM analysis, the generated coded quadratic regression model is given as follows:

(1) $$Conductivity=7.341-0.570X_{1}+0.000195X_{1}{}^{2}-0.131X_{2}-0.000080X_{2}{}^{2}+0.00131X_{1}X_{2}$$

(2) $$Resistivity=145.5-100.2X_{1}-179.0+28.7+29.3X_{2}{}^{2}-34.5X_{1}X_{2}$$

where X_1_ and X_2_ represent the coded variables in terms of sonication time and sonication power, respectively.

**Figure 3:**
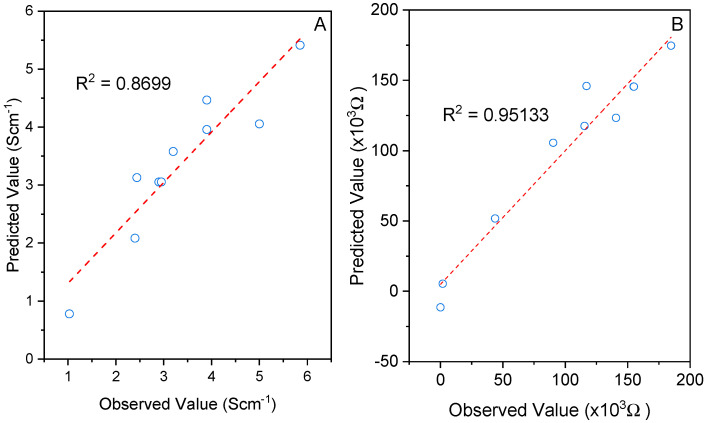
Parity plot for the observed and predicted (**A**) conductivity and (**B**) resistivity.

**Figure 4:**
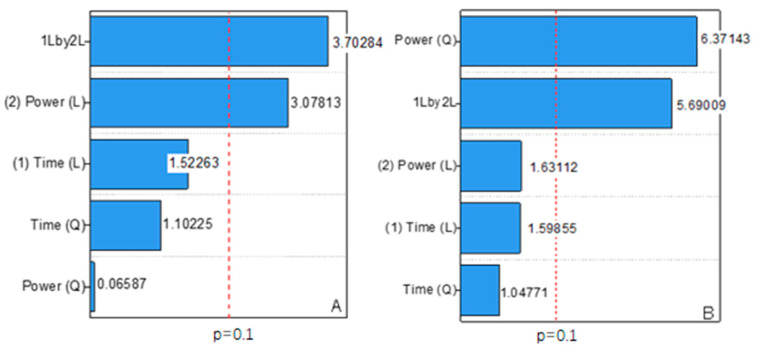
Pareto chart of (**A**) conductivity and (**B**) resistivity.

Figure 3 reports a parity plot of observed and predicted conductivity and resistivity values. The coefficients of determination (R^2^) for conductivity (Figure 3A) and resistivity (Figure 3B) were 0.8699 and 0.9513, respectively. This finding implied that the regression model can accurately explain the experimental data. Generally, the empirical model can explain the majority of variability in essay reading, which should be at least 0.75 or higher [32,33]. By numerically evaluating the model adjustment and the statistical significance of the main factors and their interactions, ANOVA is the most reliable method for assessing the quality of the fitted model. According to the ANOVA results in Table 2, the F-values for conductivity and resistivity exceeded the tabulated F-value (F0.10 = 4.05), indicating that the model acquired from Equations (1) and (2) provided a good prediction at a 10% significance level [31,34]. Figure 4 shows the t-distribution values of each model term in a Pareto chart. The interaction term for sonication time (X_1_) and sonication power (X_2_), as well as the linear term for power, prominently affected the conductivity (Figure 4A). Meanwhile, considering the resistivity values, the quadratic term for power (X_2_^2^) and the interaction term for sonication time with sonication power (X_1_X_2_) were significant at a 90% level, as evidenced by the larger t-values than the other variable terms. The remaining factors were barely significant in conductivity and resistivity because their *p*-values were greater than 0.10. Hence, this model was obviously appropriate to determine the optimum conditions for preparing PPy nanocomposites.

**Figure 5:**
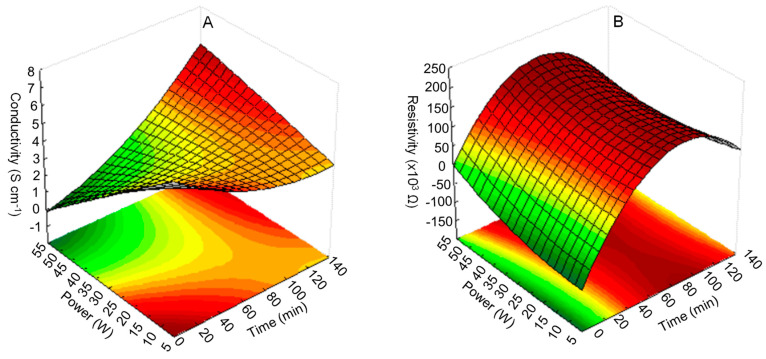
Response surface plot for response: (**A**) conductivity and (**B**) resistivity.

Response surface plots and contour line diagrams were used to determine the optimum reaction conditions and understand the relationship between variables and responses. Figure 5A depicts the combined effect of sonication power and time on conductivity. The results indicated that the maximum PPy conductivity based on the experimental data was 5.85 Scm^−1^ under the operating condition of 15 min and 10 W. Furthermore, the lowest PPy conductivity was found when the sonication time was 15 min and the sonication power was 50 W. This result was probably due to the higher power that can disturb the polymer chain of PPy and increase the resistivity. Electrical conductivity is very sensitive to morphology and crystallinity [35]. The ultrasound irradiation produced a more disordered polymer chain and increased the motion of the molecular chain. However, with increased ultrasound intensity, the nucleation rate and crystallinity were affected, resulting in decreased conductivity. Overall, sonication time and sonication power highly affected the conductivity of PPy, which can also be explained by the Pareto chart in Figure 4. The Pareto chart reported a large t-value for the interaction term of sonication power and sonication time. The relationship between power and time on resistivity is illustrated in Figure 3B. Increased sonication power slightly affected the resistivity. Conversely, PPy resistivity significantly increased with prolonged sonication time. PPy resistivity decreased slightly after reaching the maximum, indicating that prolonged polymerization led to slow hydrolysis and eventually to a high resistivity [26]. Table 3 compares the optimum operating conditions with the optimum conductivity based on experimental and predicted values. According to the experimental measurements, the PPy conductivity obtained under the optimum conditions was 2.334 Scm^−1^, whereas the model predicted 2.249 Scm^−1^. This relative error of 3.64% between the experimental and predicted data was acceptable.

### 3.2. Characterization of PPy

#### 3.2.1. Morphological Studies

**Figure 6:**
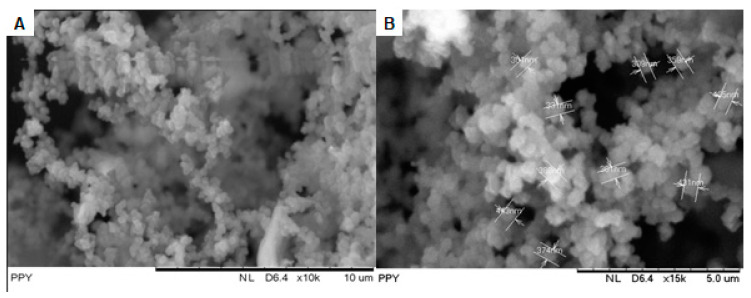
SEM images of the optimized PPy. (**A**) 10 μm; (**B**) 5 μm.

**Figure 7:**
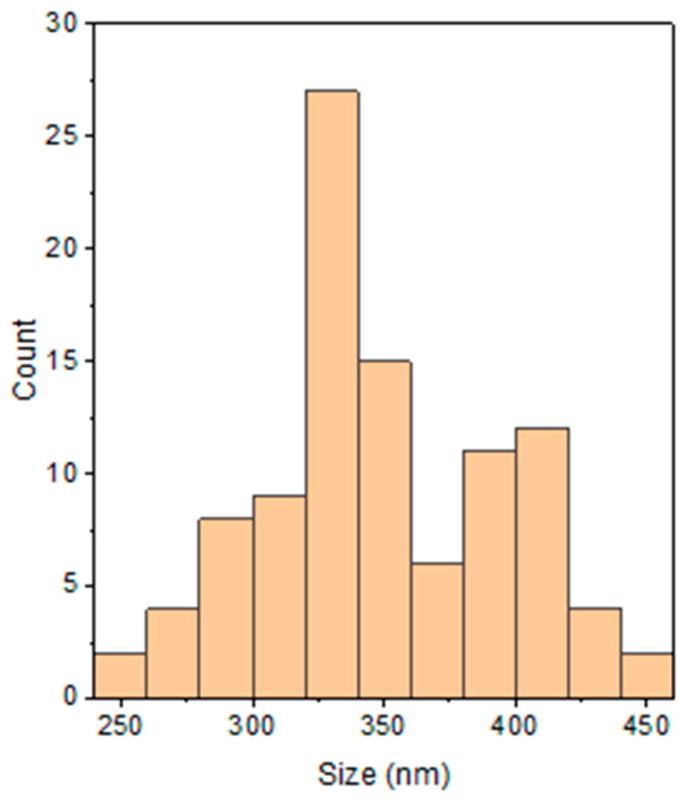
Particle size distribution interpreted from the SEM images of the optimized PPy.

Figure 6 illustrates the SEM images and particle size distribution of optimized PPy obtained using ultrasonic irradiation-assisted chemical polymerization at 10 μm (Figure 6A) and 5 μm resolution (Figure 6B) respectively. The synthesized PPy particles were perceived to be closely packed with one another and had a granular morphology, in which the globules grew one over the other and formed a continuous structure. A similar morphology has been observed by Yussuf et al. [36] and Megha et al. [37]. Moreover, the PPy particles appeared to have a more homogeneous surface structure and were thinner and more compact, with an average particle size range of granules of 325 nm (Figure 7). This phenomenon suggested that ultrasonic irradiation can significantly alter the surface structure of PPy particles owing to improvements in nucleation and growth, which produced a great number of nucleus sites at the first electro-polymerization stage. The asymmetric collapse of cavitation bubbles was the primary cause of the increased number of nucleus sites [38]. SEM analysis clearly indicated that PPy was successfully prepared with an ultrasonic irradiation power of 24 W and a sonication time of 17 min. This result was consistent with the PPy morphology reported in a previous study [39,40]. Indeed, uniform granular morphology is useful in various applications, including gas molecule sensing and absorption [41].

#### 3.2.2. FTIR Studies

**Figure 8:**
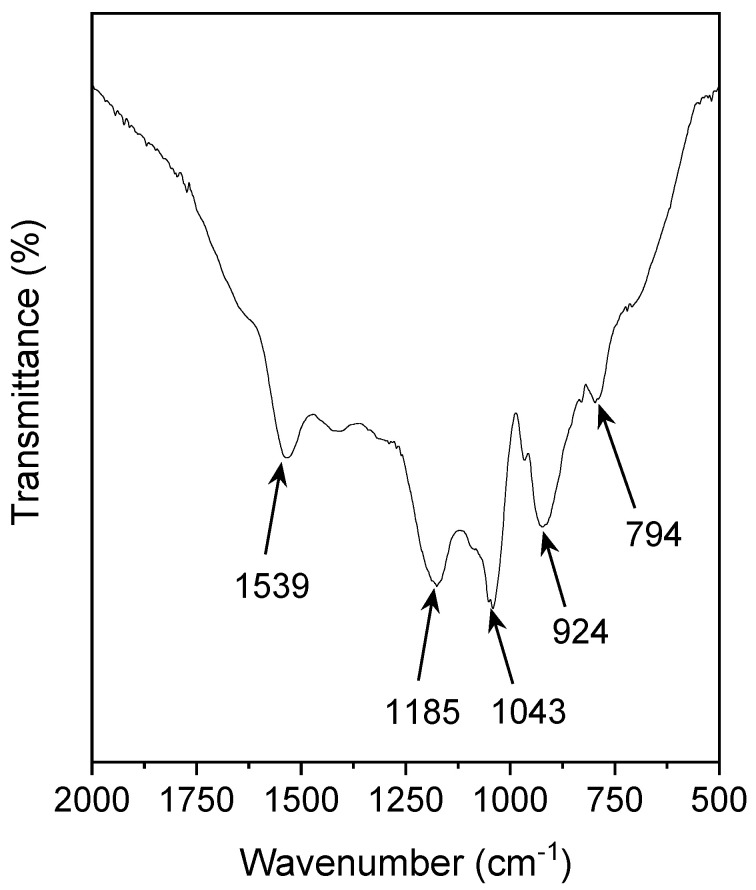
FTIR spectrum of the optimized PPy.

To verify the chemical structure of the PPy nanoparticles, PPy was then subjected to FTIR spectroscopy, and the spectrum is shown in Figure 8. No significant peaks ranged within 400−500 and 2000−4000 cm^−1^, so the spectral range was set at 2000−500 cm^−1^. The typical characteristic peaks of PPy situated at 1539, 1185, and 1043 cm^−1^ were assigned to C = C stretching vibration, C-N stretching vibration, and =C-H bending vibration, respectively [7,42]. The band at 924 cm^−1^ corresponded with the C = N^+^-C stretching vibration [42], indicating the doping of PPy with FeCl_3_ and the formation of charge carriers during synthesis. Additionally, the characteristic peaks at 794 cm^−1^ may belong to the C-H out-of-plane deformational vibration mode of the PPy ring [42,43]. The FTIR results clearly substantiated the formation of the PPy polymer and its composition structure.

#### 3.2.3. Thermal Analysis

**Figure 9:**
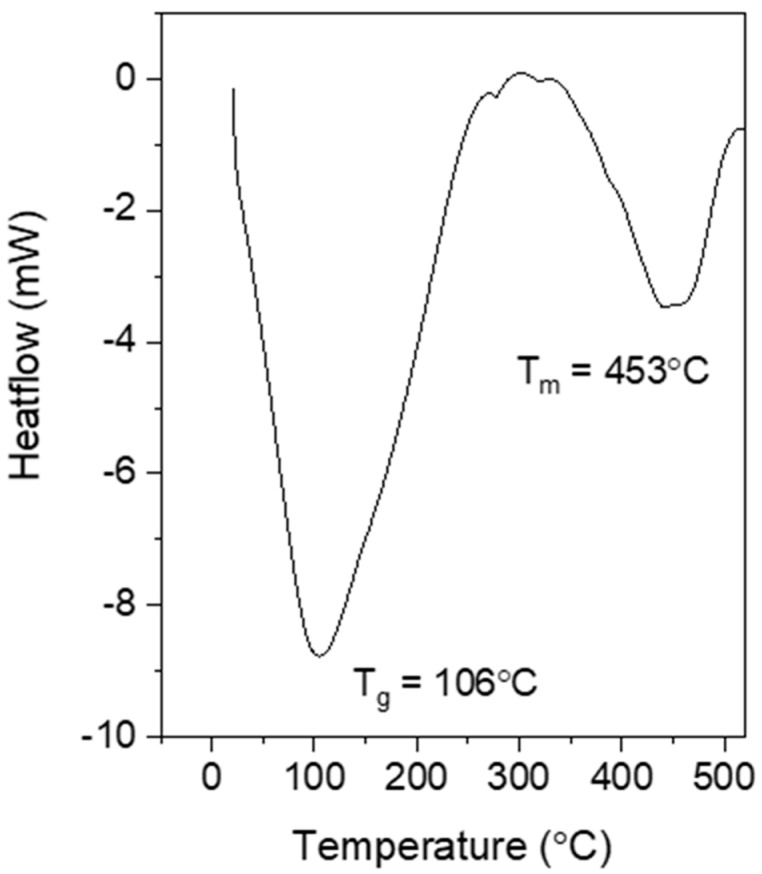
DSC results for the optimized PPy.

The thermal performance of a material is an important criterion, especially when used in electronic devices and their applications. Thermal performance depends on the material’s composition and microstructure [44,45]. Figure 9 shows the DSC curve for the optimized sample of PPy. DSC testing was used to determine the glass transition temperature (T_g_) and melting temperature (T_m_). A broad endothermic dip at 106 °C was observed, indicating the T_g_ of PPy. The T_g_ for PPy is reportedly between 100 and 120 °C [46]. Normally, the T_g_ appears as sudden changes in slopes in DSC curves, but sometimes it appears as an endothermic peak at lower temperatures [47]. A similar T_g_ of PPy has been obtained by another researcher [48]. Meanwhile, the T_m_ was observed at 453 °C. Thus, the PPy synthesized with ultrasound irradiation had good thermal stability owing to its improved morphology. Ahmad et al. [49] investigated the effect of ultrasound irradiation on the characterization of starch nanoparticles. They discovered that ultrasound irradiation disrupts the polymer crystal structure and increases the hydrocarbon chain, which may be due to the increase in Van der Waals and hydrogen bonds, in turn leading to increased T_m_. Similar results were also found by Hasanvand et al. [50], i.e., more energy is needed to melt the crystals owing to the high intermolecular interactions within them.

**Figure 10:**
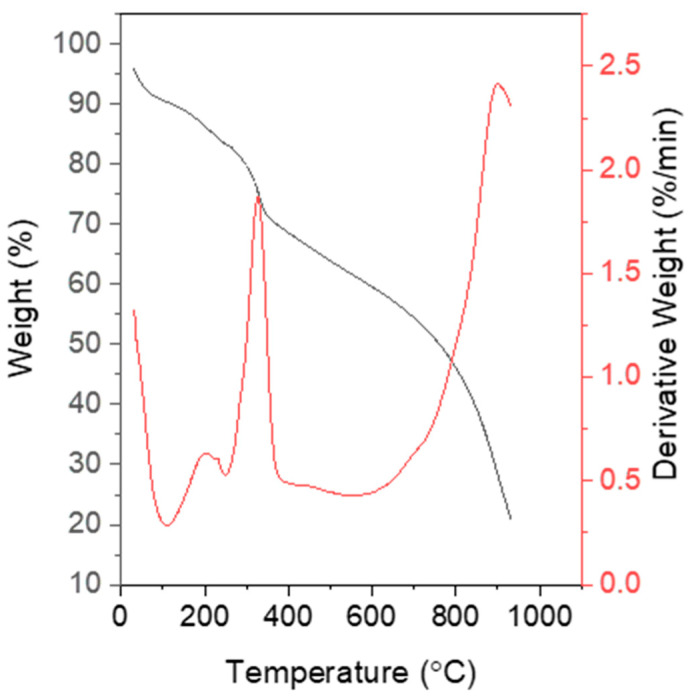
TGA and DTG curves of the optimized PPy.

Thereafter, TGA was conducted to further investigate the thermal properties of the synthesized PPy. Figure 10 reports the PPy curves under inert conditions. The first weight loss occurred at 110 °C, which was the temperature required to remove the moisture completely. The second degradation occurred at 160 °C, which could be attributed to the loss of dopant acid or possible impurities in the samples. The third stage was noted at 350 °C, corresponding with the degradation in the polymer backbone with a weight loss of 28.3% [36,47]. PPy synthesized using ultrasonication was presumed to have better thermal stability than PPy prepared using the traditional method, which was probably due to the better dispersion of the PPy nanoparticles [51]. According to Yussuf et al. [36], PPy synthesized using chemical oxidative polymerization starts to degrade at 227 °C. Meanwhile, the DTG curve shows three peaks, indicating the three stages of weight loss in PPy, in agreement with the TGA results. The first stage was the evaporation of moisture and volatile impurities, the second was the loss of dopant acid, and the third was the thermal decomposition of PPy.

## 4. Conclusions

PPy nanoparticles were successfully synthesized using ultrasonic-assisted chemical oxidative polymerization with various sonication parameters (power and time). The electrical, surface morphology, structure, and thermal stability properties of the samples were investigated. The study used RSM to optimize the electrical conductivity of PPy, revealing that its conductivity highly depended on sonication power and time. The optimized sonication parameters were found to be 17 min and 24 W, respectively, with a marginal error of 3.64% from the regression analysis. The SEM images verified that PPy exhibited a granular morphology. FTIR confirmed the existence of the PPy chemical structure and proved the successful polymerization of PPy. Moreover, DSC and TGA indicated that PPy synthesized using ultrasonic-assisted chemical oxidative polymerization exhibited high thermal stability with higher T_m_ (453 °C) and degradation temperature (350 °C). All these results indicated the great potential of PPy synthesized using this method as a conductive material for advanced electronic applications, demonstrating the significance of this study in advancing the development of conductive polymers.

## Figures and Tables

**Table 1:** Experimental design and response value.

Run	Factors		Response, Y_1_ (Conductivity, Scm^−1^)		Response, Y_2_ (Resistivity, × 10^3^ Ω)	
	Time, X_1_ (min)	Power, X_2_ (W)	Observed Values	Predicted Values	Observed Values	Predicted Values
1	15.0	10.0	5.85	5.41	0.08	−11.4382
2	15.0	50.0	1.03	0.779	43.8	51.78915
3	120.0	10.0	3.20	3.58	140.7	123.2975
4	120.0	50.0	3.90	4.47	115.4	117.542
5	15.0	30.0	2.44	3.13	1.85	5.37624
6	120.0	30.0	5.00	4.05	90.4	105.6205
7	67.5	10.0	3.90	3.96	117.0	145.9279
8	67.5	50.0	2.40	2.08	184.8	174.6638
9	67.5	30.0	2.90	3.05	154.8	145.4966
10	67.5	30.0	2.95	3.05	155.0	145.4966

**Table 2:** ANOVA.

Sources	Sum of Square (SS)	Degree of Freedom (DOF)	Mean Square (MS)	F-Value	F_0.10_
*Conductivity*					
Regression (SSR)	14.853	5	2.971	5.344	4.05
Error	2.222	4	0.556		
Total SS	17.075	9			
*Resistivity*					
Regression (SSR)	36,401.02	5	7280.204	15.638	4.05
Error	1862.22	4	465.56		
Total SS	38,263.24	9			

**Table 3:** Optimum conditions.

Process Parameters	Model-Optimized	Experimental Value
Sonication Time (mins)	17.7	
Sonication Power (W)	24.3	
Conductivity (S/cm)	2.249	2.334

## Data Availability

Not Applicable.
